# Reconstructive Challenges of Proximal Ulnar Bone Tumors: Our Experience with Biological Osteoarticular Reconstruction Using Extracorporeal Irradiation and Reimplantation

**DOI:** 10.1155/2019/7812018

**Published:** 2019-04-11

**Authors:** Manit K. Gundavda, Manish G. Agarwal, Rajeev Reddy

**Affiliations:** Orthopedic Oncology, P. D. Hinduja Hospital and Medical Research Center, Mumbai, India

## Abstract

**Introduction:**

Limb salvage surgery following proximal ulna resection poses a challenge in reconstruction of the complex elbow anatomy. Various reconstruction methods described offer inadequate restoration of function and stability. Following resection of proximal ulna tumors, we aimed to restore the joint using the resected osteochondral segment of proximal ulna treated with extracorporeal irradiation and reimplantation.

**Questions/Purposes:**

(1) Does irradiated osteoarticular autograft reconstruction for proximal ulna allow anatomical joint restoration and what are the oncological and functional outcomes? (2) Is there evidence of graft-related complications or osteoarthritis at a minimum of 2 years follow-up with irradiated osteoarticular autografts for the proximal ulna? (3) How does our method of reconstruction fare as compared to reported reconstruction options in the literature? *Materials and Methods*. 3 patients with primary bone tumors involving the proximal ulna underwent limb salvage surgery with en bloc resection and reconstruction using the resected bone after treating it with extracorporeal irradiation of 50 Gy. Minimum follow-up of 2 years was considered for assessment of final outcomes. Radiographs were assessed for bony recurrence, union across osteotomy junction, and signs of joint arthritis. Functional outcome measures included range of movement, muscle power testing, and functional and disability scores.

**Results:**

2 complete and 1 partial olecranon involving proximal ulnar resections were performed for three cases of Ewing's sarcoma in 2 males and 1 female. Follow-up ranged from 28 to 42 months, and all patients continue to remain disease free. All 3 patients have achieved full range of flexion-extension and pronosupination movement in comparison to the contralateral side. Muscle power for flexion and extension was 5/5. MSTS score: 100% (30/30); MEPS score: 100; and DASH score: zero were achieved for all patients. Union across osteotomy junctions at median follow-up of 8 months without need for intervention/bone grafting was achieved. No complications related to joint laxity/subluxation, infection, graft fracture, or implant failure was noted. None of the patients have clinical or radiological signs of joint arthritis across the irradiated articulation.

**Conclusion:**

Biological restoration of elbow anatomy using osteoarticular irradiated graft for proximal ulna reconstruction offers great joint stability and functional outcomes. Although the potential risks of infection and graft failure need to be considered, reconstruction with the size-matched radiated autograft eliminates donor site morbidity, offers a low-cost alternative to endoprosthesis, and provides outcomes superior to any other methods of reconstruction as analyzed from the literature.

## 1. Introduction

Primary bone tumors around the elbow are rare and represent <1% of all skeletal tumors [1]. Limb salvage surgery for tumors involving the proximal ulna pose a significant challenge for wide en bloc resection sacrificing the extensor musculature, supinator muscle, and risking injury to the radial nerve [2]. However, the greater challenge lies in reconstruction of the complex interplay between the multiple joints around the elbow for stability and function. Several techniques of reconstruction have been described, including radial head transposition [2–5], fibular autografts [6, 7], allograft with internal fixation, and endoprostheses [8–10]. However, these procedures are associated with complications and deficient in resulting in a stable functional joint. We aimed to restore the anatomy using the resected segment of proximal ulna treated with extracorporeal irradiation and reimplantation to achieve a stable joint with ligament reconstruction and muscle reattachments for full function.

In our small series, we asked the following questions:

1. Does irradiated osteoarticular autograft reconstruction for proximal ulna allow anatomical joint restoration and what are the oncological and functional outcomes?
2. Is there evidence of graft-related complications or joint arthritis at a minimum of 2 years follow-up with irradiated osteoarticular autografts for the proximal ulna?
3. How does our method of reconstruction fare as compared to reported reconstruction options in the literature?

## 2. Materials and Methods

Three patients with primary bone tumors involving the proximal ulna underwent limb salvage surgery with en bloc resection of the tumor following neoadjuvant chemotherapy. The resection margins were planned on pretreatment imaging (radiographs, magnetic resonance imaging, and computerized tomography scans) to ensure a layer of normal soft tissue cover and a 2 cm bony margin.

The patient was positioned in lateral position with forearm draped free. Incision over the subcutaneous border or ulna was used with inclusion to the biopsy tract that was excised along with the tumor. Ulnar neurovascular bundle was identified and protected. The medial collateral ligament, annular ligament, and triceps tendon were cut and tagged with sutures to allow reconstruction. Muscles attached to the proximal ulna were lost as margins. The radial nerve was identified and protected before cutting the supinator muscle to deliver the specimen.

The technique for ECRT that was followed was similar to what we followed for the long bones [11]. The resected segment is soaked in vancomycin solution (2 g vancomycin in 1 L of normal saline) [12] and then double plastic-wrapped over an impervious drape to maintain sterility while being transported to the radiation department for a single fraction of 50 Gy using a linear accelerator, which was delivered over 20 to 25 minutes. The tumor was not sampled or debulked prior to radiation to avoid cross contamination of the surgical field with viable tumor. The radiated bone was received on the surgery table, and the attached muscle and all visible tumors from bone were stripped off, preserving the ligament attachments for reconstruction. Debulked tumor after ECRT was sent for histopathologic examination.

The prepared graft was soaked in fresh solution of vancomycin saline before fixing it with plates to reconstruct the gap. Ligaments and capsule sutured. Triceps tendon was sutured to the olecranon with adequate tension using No. 5 (7.0 metric) polyester braided Ethibond (Ethicon Inc., manufactured in Aurangabad, India by Johnson and Johnson Pvt. Ltd.) sutures.

Adjuvant chemotherapy was started as early as postoperative day 12 once the wound had healed. Postoperative rehabilitation allowed immediate passive and active mobilization of the elbow for flexion-extension and pronosupination within the limits of pain. Gradual increase in range of movement with stretching and muscle strengthening exercises was advised. Lifting weights and loading were allowed once radiologic union was observed across the osteotomy junctions.

Follow-up was at 3 months intervals, and assessment of oncological and functional outcomes was done with thorough clinical evaluation and imaging. Minimum follow-up of 2 years was considered for assessment of final outcomes. Radiographs were assessed for bony recurrence, union across osteotomy junction and signs of joint space narrowing, osteophytes, or arthritis. Functional outcome measures included range of movement (ROM) using a goniometer, muscle power testing as per the Lovett scale, and functional and disability scores: MSTS (musculoskeletal tumor society), MEPS (Mayo elbow performance score), and DASH (disability of arm, shoulder, and hand score).

The institution waived approval for the human protocol for this study, and all investigations were conducted in conformity with ethical principles of research.

## 3. Results

**Figure 1:**
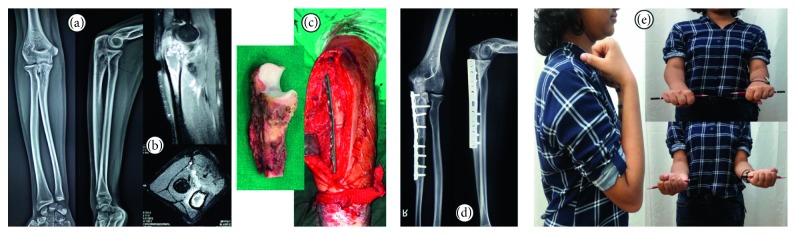
14-year-old female presented with pain in the right elbow, radiographs (a) and MRI (b) confirmed lesion in the olecranon. Biopsy proved Ewing's sarcoma and patient underwent en bloc resection and reconstruction (c) with the extrocorporeal irradiated tumor segment autograft. At latest follow-up, osteotomy junctions have united and no disease/graft- or joint-related complications seen on radiograph (d). Patient has normal range of movement and pronosupination (e).

**Figure 2:**
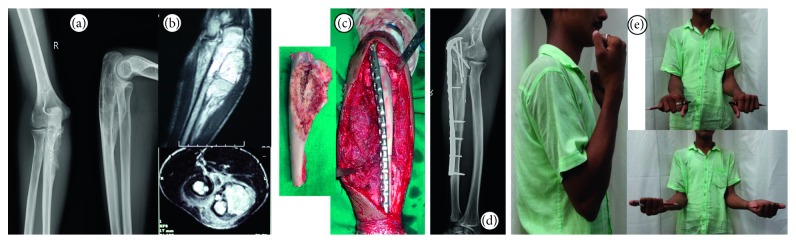
16-year-old male diagnosed with Ewing's sarcoma of the right proximal ulna presented after receiving neoadjuvant chemotherapy with radiographs (a) and MRI (b). He underwent an intercalary through the olecranon resection and reconstruction (c) with extracorporeal irradiation of resected segment. At latest follow-up of 42 months, there is no evidence of joint arthritis (d) or disease recurrence. Both osteotomy junctions have healed, and patient has excellent function (e).

Two males aged 16 and 21 years and 1 female aged 14 years (Figures 1(a) and 1(b)) with biopsy-proven Ewing's sarcoma of the proximal ulna (Table 1), following neoadjuvant chemotherapy, underwent resection of the proximal ulna with adequate gross margins. 2 complete joint-involving (Figure 1(c)) and 1 partial through-the-olecranon (Figure 2(c)) excisions underwent reconstruction with the irradiated resected segment (Figures 1(c) and 2(c)). There were no immediate postoperative wound-related complications.

With a minimum follow-up of 2 years (range 28 to 42 months), all 3 of the patients have achieved full range of movement with no restriction in pronosupination (Figures 1(e) and 2(e)). Muscle power for flexion and extension at the elbow is 5/5 on the Lovett scale. None of the patients have joint laxity, and complications such as dislocation or subluxation were absent in our series. MSTS score : 100% (30/30), MEPS score : 100, and DASH score : zero were achieved for all 3 patients. No local recurrences have been noted within the irradiated bone or in the soft tissues.

All of the four (3 ulna diaphysis and 1 proximal through the joint) osteotomy junctions have united at median follow-up of 8 months (mean 7.75 months) without need for intervention or bone grafting. No signs of joint space reduction or elbow arthritis noted at the irradiated osteochondral graft articulation.

No complications of early or late infection and nonunion, graft fracture or implant failure were seen in this series. 1 patient complaints of mild discomfort due to implant prominence over the elbow, but not wanting to undergo a surgical procedure for implant removal: no intervention done.

Our question on outcomes as reported in the literature has been addressed in the discussion and literature review.

## 4. Discussion

Reconstruction after total or partial olecranon involving proximal ulna resections for tumors is challenging. Many reconstructive approaches have been described including radial neck to humeral trochlea [2–4], free and vascularized fibula autografts [6, 7], combined fibular autograft with osteochondral irradiated graft [13], endoprosthetic reconstruction [8–10], and medialization of radius to create a single bone forearm [1]. However, each of these is limited by their shortcomings and concerns about their complications, whereas our method of reconstruction using extracorporeal radiation-treated resected segment of tumor bone aims to address most of these issues (Table 2). Extracorporeal irradiation and reimplantation is a potential biologic alternative to reconstruction where bone stock in the tumor segment is adequate; its advantages appear to be size matching (as shown in the extremities) [14–16] and low cost. However, it has not been well studied in the proximal ulna owing to the relatively low numbers of tumors involving the elbow and involvement of the articular cartilage.

The most important limitation of the study was the low number of patients and relatively short follow-up to address concerns about progressive arthritis that could arise over time. All our patients were young and were subjected to rigorous rehabilitation which may have resulted in the outcomes as we have reported. It seems likely that some of the patients will develop progressive arthritis in the autograft; however, the literature on use of radiated osteochondral autografts has not shown cartilage wear and arthritis [11]. Possibility of developing further local recurrences within the irradiated bone was analyzed and long-term data from patients undergoing extremity reconstruction have not raised any concerns. Fatigue failure is another concern over long-term follow-up and for all these reasons; these patients should receive continued follow-up. A limitation of the procedure was inability to assess margins microscopically in the resected tumor segment, as well as inability to assess response to neoadjuvant chemotherapy. Therefore, accurate planning of resection margins and assessment of metabolic response was assessed on MRI and PET scans, respectively.

Transposition of radial neck to humeral trochlea resulted in joint instability [2, 4], limitation of movement [5], and muscle weakness [3], whereas our technique allowed us to recreate the stable elbow anatomy and physiological musculoligamentous attachments for restoration of function.

Fibular autografts were associated with donor site morbidity such as leg edema [6] and joint instability [6, 7] at the fibula-humerus articulation. Long microvascular surgical procedure was performed if live vascularized fibula was used. The fibula is not size-matched for the ulnar junction, unlike the radiated autograft which fits perfectly well to reconstruct the defect.

Modular [8] and custom [10] elbow replacement endoprostheses provide an anatomical reconstruction option for joint stability [9]. However, joint replacement was associated with triceps muscle weakness [10], periprosthetic lysis, and stem loosening, resulting in revision surgeries [8–10]. Custom prosthesis is expensive, while the resected tumor bone is a cost-effective alternative and perfectly size-matched and osteointegratable scaffold eliminating prosthesis-related complications [11].

Medialization of radius to a preserved segment of proximal ulna is a resection as well as a reconstruction challenge [1]. Possibility of this procedure is only in very select few cases where it is possible to preserve the olecranon segment without compromising the tumor margins. This method may achieve a stable single bone forearm, but leads to limb length discrepancy and limited pronosupination movement [1] which was not seen in this series with our method of reconstruction.

We were limited in our assessment of the elbow after reimplantation to plain radiographs. Information from sophisticated imaging may be of great value to assess cartilage status and joint arthritis. Although we feel this procedure offers the benefits of elbow function preservation (as compared with radial head transposition) and joint stability (as compared to fibula autograft) while reducing risks associated with alternative reconstruction methods (e.g., endoprostheses), future studies with comparative data are needed.

## 5. Conclusion

Biological restoration of elbow anatomy using osteoarticular irradiated graft for proximal ulna reconstruction offers great joint stability and functional outcomes. Although the potential risks of infection and graft failure need to be considered, reconstruction with the sized-matched radiated autograft eliminates donor site morbidity, offers a low-cost alternative to endoprosthesis, and provides outcomes superior to any other methods of reconstruction as analyzed from the literature.

## Figures and Tables

**Table 1:** Patient demographics and outcomes.

Patient	Age	Sex	Diagnosis	Date of surgery	Resection length	Joint resection	Fixation	Follow-up	ROM	Osteotomy junction (time to union)	Joint status
1	14	F	Ewing's sarcoma	June 2016	8 cm from tip of olecranon	Complete	3.5 mm LC-DCP	2 years 4 month	Full	United (8 months)	Normal
2	16	M	Ewing's sarcoma	April 2015	Intercalary	Partial: 2/3^rd^ articular surface resected	3.5 mm precontoured locking plate	3 years 6 months	Full	United (proximal: 6 months Distal: 8 months)	Normal
3	21	M	Ewing's sarcoma	January 2016	6.5 cm from tip of olecranon	Complete	3.5 mm precontoured locking plate	2 years 10 months	Full	United (9 months)	Normal

**Table 2:** Comparison of outcomes of various reconstruction options reported in the literature, following proximal ulna resection for bone tumors.

Study, year	Cases	Reconstruction technique	Results	Complications and remarks
Current study	3	Osteoarticular extracorporeal irradiation and reimplantation of proximal ulna resected segment	ROM: 0° to 130° Pronosupination: full Power: 5/5 MSTS: 100% (30/30) DASH: zero MEPS: 100	Implant prominence over the elbow in 1 case
Rydholm, 1987 [3]	1	Radius neck to humerus trochlea articulation	ROM: 35° to 135° Pronosupination: 40°	Muscle weakness nearly 50 percent of normal
Gianoutsos et al., 1994 [6]	1	Osteocutaneous fibular free flap	ROM: 10° to 100° Pronation: 45° Supination: 35° Power: 4/5	Instability of the joint Donor site: leg edema
Kimura et al., 2002 [7]	1	Vascularized fibular graft	MSTS 100% (30/30)	Annular ligament reconstructed for joint stability
Weber et al., 2003 [8]	11 elbows (1 proximal ulna tumor)	Total elbow replacement	Mean MSTS: 83% (25/30)	Periprosthetic lysis
Duncan et al., 2008 [2]	2	Radial neck to humerus trochlea transposition	MSTS (Mean): 88.3% (26.5/30)	Joint instability and muscle weakness
Guo et al., 2008 [9]	19 elbows (5 proximal ulna tumors)	Total elbow arthroplasty	MEPS: Good in 14/19: 77.8% Poor in 4/19: 22.2%	Stem loosening Periprosthetic lysis Revision surgeries
Ogose et al., 2010 [13]	1	Combined vascularized fibula + osteochondral extracorporeal irradiated graft.	ROM: 20° to 120° Pronation: 80° Supination: 10°	Proximal osteotomy site nonunion: bone grafting at 16 months after surgery
Chen et al., 2012 [4]	1	Radius neck to humerus trochlea transposition	MSTS: 83% (25/30) ROM: 10° to 90°	Joint instability Muscle strength weakness
Sewell et al., 2012 [10]	4	Custom proximal ulna endoprosthetic replacement	Mean MSTS: 90% (27/30) Mean TESS: 81 (73 to 88)	Triceps weakness
Sulko, 2013 [5]	1	Radial head transposition with inverted V-plasty of triceps	MSTS: 96.67% (29/30) DASH: zero Power: 5/5	Restricted Pronation. Limb length discrepancy: 2 cm
Puri et al., 2016 [1]	1	Medialization of radius to a preserved proximal articular segment of ulna	ROM: 10° to 130° MSTS: 90% (27/30)	Restricted pronosupination Limb length discrepancy. Implant prominence over elbow

ROM: range of movement. MSTS: musculoskeletal tumor society score. MEPS: Mayo elbow performance score. TESS: Toronto extremity salvage score. DASH: disability of arm, shoulder, and hand score. Muscle power as measured on the Lovett scale.

## Data Availability

The data used to support the findings of this study are available from the corresponding author upon request.
